# *“I Was Told I Needed Both Knees Surgically Replaced, but I Walked the Camino Trail Instead.”* A Case Report of A 66-year-old Male with Knee Osteoarthritis

**DOI:** 10.1080/24740527.2026.2717606

**Published:** 2026-09-29

**Authors:** Erin MacIntyre, Tasha R. Stanton, Peter Ninnes, Kim Wilson, David S. Butler, Felicity A. Braithwaite

**Affiliations:** a IIMPACT in Health, School of Allied Health and Human Performance, College of Health, Adelaide University (AU), Adelaide, Australia; b Persistent Pain Research Group, Hopwood Centre for Neurobiology, Lifelong Health Theme, South Australian Health and Medical Research Institute (SAHMRI), Adelaide, Australia; c Co-researcher for the Persistent Pain Research Group (AU), Adelaide, Australia; d Adelaide West Physiotherapy, Adelaide, Australia

**Keywords:** Osteoarthritis, case report, pain science education, knowledge mobilization

## Abstract

**Background:**

This case report describes the lived experience of a 66-year-old male (PN) with bilateral knee osteoarthritis whose engagement with best practice conservative management resulted in a positive outcome of unanticipated scope/magnitude. PN had long-term symptomatic knee osteoarthritis and was advised that bilateral arthroplasty was required. He chose to avoid surgery, instead participating in a clinical trial evaluating the effect of embedding pain science education (PSE) into an individualized, graded exercise program (EPIPHA-KNEE trial).

**Methods:**

The intervention involved four weekly in-person sessions, five telehealth sessions (weeks 5–8, 12), and two top-up in-person sessions (weeks 21, 39). A key intervention component was activity-related goal setting, with cognitive reconceptualization and activity progression underpinned by PSE. PN set an “aspirational goal” – to walk the Camino de Santiago Trail. Clinical outcomes and device-measured activity levels were assessed at baseline, 8, 26 and 52 weeks.

**Results:**

Relative to baseline, PN experienced improvements in knee pain (83% reduction), function (71% improvement), and activity levels (50% increase) at 52 weeks with cessation of pain medication use and resolution of knee swelling and night pain. PN experienced continuing benefit, walking 320 km of the Camino Trial in 14 days one year after trial completion.

**Conclusion:**

PN’s achievement of his aspirational goal is an exemplar of the unanticipated scope of osteoarthritis improvement with best-practice non-surgical management and is shared via a video interview. Documenting and sharing such inspirational stories with people living with knee osteoarthritis may prove an important source of hope and highlight the change potential for non-surgical treatment.

## RÉSUMÉ

**Contexte :** Ce rapport de cas décrit l’expérience vécue par un homme de 66 ans (PN) atteint d’arthrose bilatérale du genou, chez qui une prise en charge conservatrice conforme aux meilleures pratiques a produit des résultats positifs d’une ampleur inattendue. PN présentait depuis longtemps une arthrose symptomatique des deux genoux et avait été informé qu’une arthroplastie bilatérale était nécessaire. Souhaitant éviter l’intervention chirurgicale, il a plutôt participé à un essai clinique visant à évaluer les effets de l’intégration d’une éducation aux sciences de la douleur à un programme d’exercices individualisé à progression graduelle (essai EPIPHA-KNEE).

**Méthodes :** L’intervention comprenait quatre séances en personne, à raison d’une par semaine, cinq séances en télésanté (semaines 5 à 8 et 12) et deux séances de renforcement en personne (semaines 21, 39). L’un des éléments clés de l’intervention consistait à établir des objectifs liés à l’activité. La reconceptualisation cognitive et la progression des activités reposaient sur l’éducation aux sciences de la douleur. PN s’est fixé un « objectif ambitieux » : parcourir à pied le chemin de Saint-Jacques-de-Compostelle. Les résultats cliniques et les niveaux d’activité mesurés au moyen d’un dispositif ont été évalués au début de l’étude, puis aux semaines 8, 26 et 52.

**Résultats :** Par rapport aux valeurs initiales, PN présentait, à la semaine 52, une diminution de 83 % de la douleur au genou, une amélioration de 71 % de la capacité fonctionnelle et une augmentation de 50 % du niveau d’activité. Il avait également cessé de prendre des antalgiques, et le gonflement du genou ainsi que les douleurs nocturnes avaient disparu. PN a continué de bénéficier de ces améliorations : un an après la fin de l’essai, il a parcouru 320 km du chemin de Saint-Jacques-de-Compostelle en 14 jours.

**Conclusion :** La réalisation par PN de son objectif ambitieux illustre l’ampleur inattendue des améliorations qui peuvent être obtenues grâce à une prise en charge non chirurgicale conforme aux meilleures pratiques. Son expérience est présentée dans un entretien vidéo. Le fait de documenter et de communiquer de tels récits inspirants aux personnes atteintes d’arthrose du genou pourrait constituer une source importante d’espoir et mettre en évidence le potentiel d’amélioration qu’offre la prise en charge non chirurgicale.

## Introduction

Knee osteoarthritis (OA) can cause severe pain and disability that interferes with an individual’s ability to enjoy life.^1^ Exercise is a core treatment for knee OA: it improves pain, disability, and quality of life^2^; it can delay or even prevent knee replacement surgery^3^; and, it is recommended for everyone with OA, regardless of age or OA severity.^4^ All clinical guidelines recommend exercise as a first-line treatment for OA (alongside education and weight management), with surgery considered only after conservative options have been exhausted.^5^ However, most people with knee OA do not maintain, or even attempt, an exercise program that could enable them to achieve these outcomes.^6^ One reason is the common misconception that activity or exercise may be detrimental to a “*damaged*, *degenerated*” joint (increase *“wear and tear”)*.^7–9^

Inaccurate beliefs about OA and its management are often reinforced by health professionals, who commonly describe OA as an “*unchangeable, degenerative disease.”*^8^ In Australia, people with knee pain/OA are referred to orthopedic surgeons three times more often than to non-surgical care.^10^ Knee imaging is common: it is ordered in over half of new knee OA cases to help determine whether surgery is needed, despite guidelines advocating against using imaging for this purpose.^10^ Indeed, only weak associations between radiographic structural degeneration and knee OA symptoms exist^11,12^ (although see here for instances of greater association^12,13^). Most telling perhaps, are findings showing the presence of radiographic change in the absence of pain.^14^ Regardless, the clinical sequelae of referrals based on imaging can be harmful and may contribute to Australian arthroplasty rates that are 73% higher than the OECD average.^15^ People with OA are often presented with radiographic evidence that surgery is urgently required, even though imaging findings are more likely related to their age than to their symptoms.^11^ A diagnosis and explanation of OA based on radiographic findings has been shown to increase the belief that surgery is necessary^16^ and is no longer supported by clinical practice guidelines.^17^

Another barrier to non-surgical management of knee OA is the widespread belief, held by both people with knee OA and health professionals, that conservative treatment is not a ‘cure,’ but rather a temporary management strategy that, at best, delays inevitable surgical intervention.^18,19^ However, the maximal therapeutic potential of non-surgical OA management has yet to be elucidated; indeed, OA recovery *without surgical intervention may be possible*. If true, the prevailing belief that non-surgical care only maintains current function could result in an artificial treatment ceiling effect, limiting the full potential of this management strategy. There is merit in providing evidence for the transformative effects of non-surgical management of OA to change the way this care is perceived.

The overall aim of this clinical note is to report qualitative and clinical outcomes of a participant (PN) receiving a pain science education (PSE) and exercise intervention. We also provide clinician and patient resources to facilitate intervention replication. Our work with OA consumers supports storytelling as a powerful strategy to inspire behavior change because *“personal connections can undo misconceptions”* and *“there is nothing like someone else’s experience with a good outcome – it shows it can work.”^20^* Case studies may be useful clinical exemplars to help tackle misconceptions about knee OA and provide hope to people with knee OA by demonstrating the real-world change potential of non-surgical management. Thus, we co-created a video in partnership with PN where he tells his story of recovery and demonstrates the power of conservative management of knee OA.

## Methods

This case study is reported consistent with the CARE Guideline, see Supplementary File 1 for full checklist. Ethical approval for this case report was obtained (UniSA Human Research Ethics Committee, protocol number: 202377), and PN gave written consent, including consent to disclose his story and identity.

### Case description

At the time of this case study, PN was 66 years old. He has a degree in agricultural science and worked in sustainable development internationally for 25 years. PN has a sporting background and understands the importance of exercise and muscle strengthening exercise, especially in the context of injury rehabilitation. He first noticed knee symptoms in 2009 after a game of recreational tennis, and over the following 8 years, he continued to have worsening knee pain and swelling. In 2017 PN met with a surgeon and was diagnosed with severe bilateral knee OA (Kellgren-Lawrence Grade 3 and 4 on his right and left knees, respectively) and was advised that he would require knee arthroplasty. PN recalls that the diagnosis of OA was based on imaging results, which enforced a conceptualization of OA as a structural entity, *“I looked at the x-rays and I can see more or less what is going on …. the specialist pointed out some areas that might be problematic and I looked at those areas and I thought well this is someone who has expertise in this area and they must be problematic.”* The consultation only focused on surgical options, PN described the encounter as “*you go through the consultation with all of the x-rays, you see all of these anatomical models with knee joints with red marks all over them. And then you start to think, oh this is not so good, I really don’t feel that great. And the outcome of that consultation was come back, and we will replace one or both of you knees either partially or fully. Apart from that, there was virtually no discussion about other possibilities that there might be. I came away from that appointment with more questions than answers.”*

After the appointment with the surgeon, PN decided against surgery. This decision was partly based on his previous work experience, where PN found that technological “silver bullet” solutions rarely result in optimal outcomes. “*[Surgery] is kind of a one-track way of thinking … it’s a silver bullet solution … and I thought that there has to be a more wholistic approach to how I manage this.”* PNs decision was also informed by the experiences of those around him, particularly from a friend who had poor outcomes after his knee replacement surgery. “*He went in to have his knee re-done and has had no end of troubles ever since. He has had 3 or 4 surgeries, he’s been in and out of hospitals, his ability to exercise and to move has been really impacted. It’s a really sad story.”* After making his decision, PN continued to self-manage his knee pain, with limited success for several years. *“I found that I was waking up at night because of my sore knee. I was taking pain relief [medication]. And it just become a habit … I started to think this isn’t sustainable, I can’t keep doing this.”*

### Intervention

PN enrolled in the EPIPHA-KNEE Trial in 2021 (trial registration number: ACTRN1262000104194) as a pilot participant and was allocated to receive the PSE intervention with graded aerobic and strengthening exercise. A detailed description of the PSE intervention is reported in the published protocol^21^; minor deviations for PN are reported in Supplementary File 2. In brief, PN attended 11 physiotherapy sessions over a 9-month period and was provided with a comprehensive PSE curriculum and individualized graded exercise program, with walking and lower limb strengthening components. The PSE curriculum was supported by a trial-specific version of The Knee OA Handbook (Noigroup, Adelaide, Australia), which acted as a learning reference and activity manual to reinforce educational concepts covered within physiotherapy sessions. The walking program incorporated principles of graded activity and pacing, including pre-planning walks (time/date) and gradual increases over time, and encouraged completing planned walks even when PNs knee was painful (disrupting associations between pain and movement/activity). The lower limb strengthening program was guided by similar principles (individualization, graded progression), and consisted of body weight-bearing exercises (e.g., squats, step-ups) and was progressed through the first 13 weeks of the trial.

Table 1 provides a curriculum for the PSE program PN underwent and provides links to educational resources that can supplement The Knee OA Handbook. All target concepts were covered in the first 4 sessions, with subsequent sessions largely focused on refining PN’s understanding and progression through the graded exercise program. Delivery of the target concepts was iterative (i.e., refined over multiple sessions) and involved reciprocal storytelling and metaphors. Further details of how PSE concepts were delivered is reported in the results section.

**Table 1.** PSE target concepts, resources, and strategies.

EPIPHA-KNEE Target Concept	Educational Resources, Content, and Strategies	Application within the case study	
		Important concepts for PN	PN reflection
**1. OA is a whole-body process involving systemic and local inflammation**. *“There is a new view of OA out there “*	**The Knee Osteoarthritis Handbook**: Old and new view of knee OA: pgs. 8–9 The 3 Critical Ingredients: pgs. 10–11 Power up your knowledge of inflammation: pgs. 18–22 Our Top 10 Anti-inflammatory Tips: pg. 23 Power up your knowledge about your knee: pgs. 24–26 Bone on Bone Bob: pg. 27 Exercise is great for EVERYONE’S OA knees: pg. 58 **Reflections and observations** Are there any gut links to your pain? Do you have pain in any other areas? When you’re sick (like with the flu, i.e inflamed) does your knee hurt more? Do anti-inflammatories help you? Does your pain have good days and bad days? What sort of things make your knee feel better? Has your body changed since your knee problem started? **Additional resources**: Michael Mosely inflammation and gut biome: https://www.abc.net.au/listen/programs/conversations/conversations-michael-mosley/9867346 Inflammation and diets: https://theconversation.com/9-signs-you-have-inflammation-in-your-body-could-an-anti-inflammatory-diet-help-210468 Mr Cookfulness: https://www.cookfulness.co.uk/ Flippin Arthritis (Flip the way you think about OA): https://www.youtube.com/watch?v=Wgne_9w-imA&list=PLxUVmk20As7uvNWWcbA0kjsQa5kJCOoqz	The role of systemic inflammation on knee OA symptoms was discussed in Session 2. PN and the physiotherapist discussed links between inflammation, body weight, and diet. This concept was revisited in Sessions 3 and 4. Overall, PN connected Target Concept 1 to his work in agricultural researcher, where the integrated management of an issue is more complex and demands more management/time however it also provides a more sustainable solution. Conceptualizing OA as an emergent process (i.e., not linear, multiple paths to improvement) represented an important shift in PN’s journey.	*“What I was missing was a broader understanding of what’s involved in joint pain and osteoarthritis …. Because once we’re given options, we can make more informed decisions about what we choose to do.” “My professional background helped me think about my personal situation with my osteoarthritis. Many of the solutions in developing countries are a whole package of things …. And so I came to realise that technology isn’t a silver bullet. This got me thinking about my own situation for osteoarthritis. I was thinking well, the solution to this is not necessarily going to be surgery.”*
**2. The body has multiple protective processes including pain** *“The purpose of pain is protection”*	**The Knee Osteoarthritis Handbook**: Dave’s drug cabinet lesson: pg. 30 Scans, pains and knee OA: pg. 31 The modern pain story: pgs. 32–33 What’s that noise?: pgs. 34–35 **Reflections and observations** 50% of people with OA don’t know they’ve got OA When you’re in pain, are there any other protective systems in action? Can my knee feel stiff without being stiff? Most of the world believes there are pain endings, we’ve just challenged this If there are no pain detectors, why do I hurt? **Additional resources**: Drug cabinet in the brain: https://www.youtube.com/watch?v=Gd2NaGZa7M4&t=20s Smudging: https://www.youtube.com/watch?v=3QVAY5stO3U&t=64s Pain is a protector (Pain Revolution): https://painrevolution.org/pain-facts/ Key messages about pain (Flippin Pain): https://static1.squarespace.com/static/660e7f602ad163564c7ce82a/t/685d0be215ec7e2aa91ef14b/1750928360146/Key+Messages+Leaflet+DIGITAL.pdf Pain in the brain: https://www.abc.net.au/listen/programs/healthreport/the-brains-role-in-pain/7735610	In Session 1, PN and the physiotherapist discussed the modern pain story (i.e., peripheral and central nervous system changes associated with persistent pain). In Session 2, a discussion regarding how knee OA is diagnosed (i.e., clinical diagnosis is now recommended by clinical practice guidelines), challenged PN’s understanding of OA and the completeness of his previous diagnosis of knee OA. Together, this gave PN ‘permission to move.’ In Session 4, PN brought his x-rays to the appointment. The physiotherapist challenged views of OA knees being damaged/deficient, instead reinforcing positive views towards his knees by suggesting that PN should frame them and put them on his wall. Having this validation helped PN believe that his aspirational goal of walking the Camino de Santiago was possible. Discussing swelling as a protective response was revisited in all sessions. The reduction in swelling after the first session was a powerful example of experiential learning. PN saw an immediate change in knee swelling, long before any structural joint changes could have occurred. This reinforced his updated understanding of the difference between knee structures and protective symptoms.	*“While I was on the physio program, particularly in the first 3 or 4 weeks, I had a couple of occasions when I had some swelling. I knew that wasn’t a bad thing, it was my body telling me that, okay maybe I have slightly overdone it the day before, so I reduced my load for a day or two before going back to full load mode again. And that was fine.” “I know that pain is a normal thing …. The education program taught me about pain and how we can raise our expectations in terms of managing pain. And I have certainly been able to do that. Frankly it [PN’s knee pain] doesn’t seem to bother me.” “Having a good support team, having access to good HCPs and simply enjoying what I was doing and knowing that it was making my life better … it was a whole combination of factors that came together [to help my recovery]”*
**3. Pain and other protective processes are influenced by bio-, psycho- and social contexts**. *“Pain is completely dependent on context”*	**The Knee Osteoarthritis Handbook**: Scans, pains and knee OA: pg. 31 The modern pain story: pgs. 32–33 Why does my knee pain vary?: pg. 36 Your personal knee pain formula: pg. 39 DIMS/SIMS: pgs. 40–41 Protectometer: pgs. 42–51 Twin peaks: pgs. 52–53 DIM/SIM sweet zone drivers: pg. 63 Stepping out menu (many options!): pgs.66–80 **Reflections and observations** Why does your pain have good and bad days? Reflections on the protectometer DIMs and SIMs hide in hard to find places Have your DIMs and SIMs changed sides? Reflect on context and the notion of network Reflect on examples of how context might alter other protective systems (eg breathing) **Additional resources**: Lorimer Moseley snake story: https://www.youtube.com/watch?v=gwd-wLdIHjs Twin peaks video: https://vimeo.com/449983911; password: Epiphany. Twin peaks fact sheet: https://drive.google.com/file/d/14trBBgY7szz0dHl-Ho9PylkFBkOfAuvA/view Drug cabinet in the brain: https://www.youtube.com/watch?v=Gd2NaGZa7M4&t=20s Tale of Two Nails: https://www.noigroup.com/noijam/a-tale-of-two-nails/	DIMS/SIMS were briefly discussed in Session 2, after PN brought up that his knees were more painful when the weather was bad. The concept of DIMS was briefly touched on in Session 3 and 4, but was less relevant to PN. In Session 4, PN determined that he would walk the Camino de Santiago. This SIM was a motivator, and the excitement of achieving his aspirational goal added positive context to his walking program. PN’s aspirational goal was an important SIM – impacting not only walking, but other aspects in his life (i.e., researching travel plans, conversations with friends/family)	*“I did find a DIM – walking in the city …. One of the things that annoys the hell out of me is traffic. So I found that walking, even in a relatively quiet part of the city, with traffic was certainly a DIM for me.” “There were some other aspects of DIMS and SIMS that I did …. I spent one day a week walking on the Encounter walkway with my backpack loaded down with about 10 to 12 kilograms of stuff …. once I had it on and was walking away and at peace with the world I really didn’t notice it.”*
**4. Humans are bioplastic**	**The Knee Osteoarthritis Handbook**: Cartilage needs you to move to improve: pg. 26 Power up knowledge about the knee in your brain: pgs. 28–29 Twin peaks: pgs. 52–53 Exercise is great for EVERYONE’S OA knees: pg. 58 Your activity sweet zone: pgs. 59–62 DIM/SIM sweet zone drivers: pg. 63 **Reflections and observations**: Personal bioplastic changes: E.g. how scars heal, or *‘Have you ever improved your fitness – what changes could you see and feel?’* Your own changes to your Activity Sweet Zone during the programme so far Discuss on motivation and barriers to maximal bioplastic potential Can you lift your expectations of knee future? **Additional resources**: Bioplasticity (noijam): https://www.noigroup.com/noijam/bioplasticity/ Bioplasticity (video): https://painhealth.com.au/pain-module/neuroplasticity/ A new way to think about pain (Lauren Cannell TEDTalk): https://www.youtube.com/watch?v=ruKVTOK8gTg	Session 2 involved a long discussion about cartilage. This included how busting the ‘bone-on-bone’ myth and how exercise can improve cartilage health. PN immediately took to understanding bioplasticity in the periphery, particularily regarding knee structures. This then paved the way for understanding bioplastic changes in the central nervous system (i.e., Target Concepts 1 and 2)	*“We often hear about [knee OA as being] bone-on-bone … which conjures up images of grinding bones. it’s all quite negative …. So instead, the concept of doing exercise, particularly load-bearing exercises, in order to have healthy cartilage really appealed to me”*
**5. Targeted and graded OA knowledge, inflammation management, and contextual physical activity are essential OA treatments**	**The Knee Osteoarthritis Handbook**: The 3 Critical Ingredients: pgs. 10–11 Steps to recovery: pgs. 12–14 The method: pgs. 15–17 Twin peaks: pgs. 52–53 Exercise is great for EVERYONE’S OA knees: pg. 58 Your activity sweet zone: pgs. 59–62 Stepping out menu (many options!): pgs.66–80 Knee OA walking program: pgs. 82–83 Knee oilers: pgs. 84–90 **Reflections and observations**: Achieving and reflection on competencies of each component Reflection on goal achievement, goal setting and goal adjusting Problem solving various ways of treatment **Additional resources**: Setting SMART goals: https://livewellwithpain.co.uk/resources-for-people-with-pain/shortcuts/make-your-goals-smart/ Setting goals to stay active: https://livewellwithpain.co.uk/resources-for-people-with-pain/ten-footsteps-to-living-well-with-pain/setting-goals/ Flippin Arthritis (Is movement really right for me?): https://www.youtube.com/watch?v=m0zrrqzVpXc	In Session 1, the physiotherapist introduced the graded walking program. This was re-visited in all subsequent sessions, which focused on graded increases of walking capacity. This Target Concept aligned with PN’s goals – he enjoyed physical activity and wanted to become more active. Implementation of the walking program was therefore highly personally relevant to PN.	*“That [the education program] was really the beginning of the realisation of the shift of seeing my knees as a liability to seeing them as an asset. Which I think is important because it acts as a springboard.”*
**6. Effective education requires knowledge building skills**. *‘Knowledge is the greatest pain liberator of all’*	**The Knee Osteoarthritis Handbook**: Steps to recovery: pgs. 12–14 The method: pgs. 15–17 Stepping out menu (many options!): pgs.66–80 **Reflections and observations**: Deep reflection – have you ever been taught how to learn? You can be the expert on your OA/pain -Building personal expertise in OA pain Have you shared any of this information with your loved ones? Do you agree or disagree with the information you’ve been given? What did you take away from the last session? What have your learnt here that you may be able to use in the future? **Additional resources**: Learning to learn: https://vimeo.com/449981182, password: Epiphany Developing a growth mind-set (Carol Dweck TEDTalk): https://www.youtube.com/watch?v=hiiEeMN7vbQ Learning doesn’t happen to you: https://onething.painsci.org/learning-doesnt-happen-to-you-lorimer-moseley-one-thing/	In session 1, PN connected this target concept to his work as a scientist – where he learns from others and considers problems from multiple perspectives. PN enjoyed reading the handbook (finished it by Session 4). He valued discussing concepts with the physiotherapist and his family.	*“My professional background has taught me …. If I don’t really understand what’s going on or want to look for other options I know how to do that.” “In a development context in developing countries it is important to engage with beneficiaries (‘consumers’) since indigenous knowledge (lived experience) is a vital part of any program”*

**Additional resources** were not provided to PN during the case study, rather they have been provided as alternatives for people who cannot access the Knee Osteoarthritis Handbook. The resources have been reviewed by PSE experts and align with the intervention than PN received.

### Outcomes and analysis

In 2024, we video recorded an interview with PN during which he told his knee OA story and experiences with the trial. The semi-structured interview guide is contained in Supplementary File 2. We worked with PN to co-create a brief 4-minute video capturing his inspirational story (Figure 1), with the aim of converting this into an education resource in an accessible format for people living with OA and health professionals. PN and members of the research team (EM, FAB, and TRS) reviewed all video content multiple times and collaboratively selected quotes that captured key moments of PN’s experience to provide context to the case report

**Figure 1.**
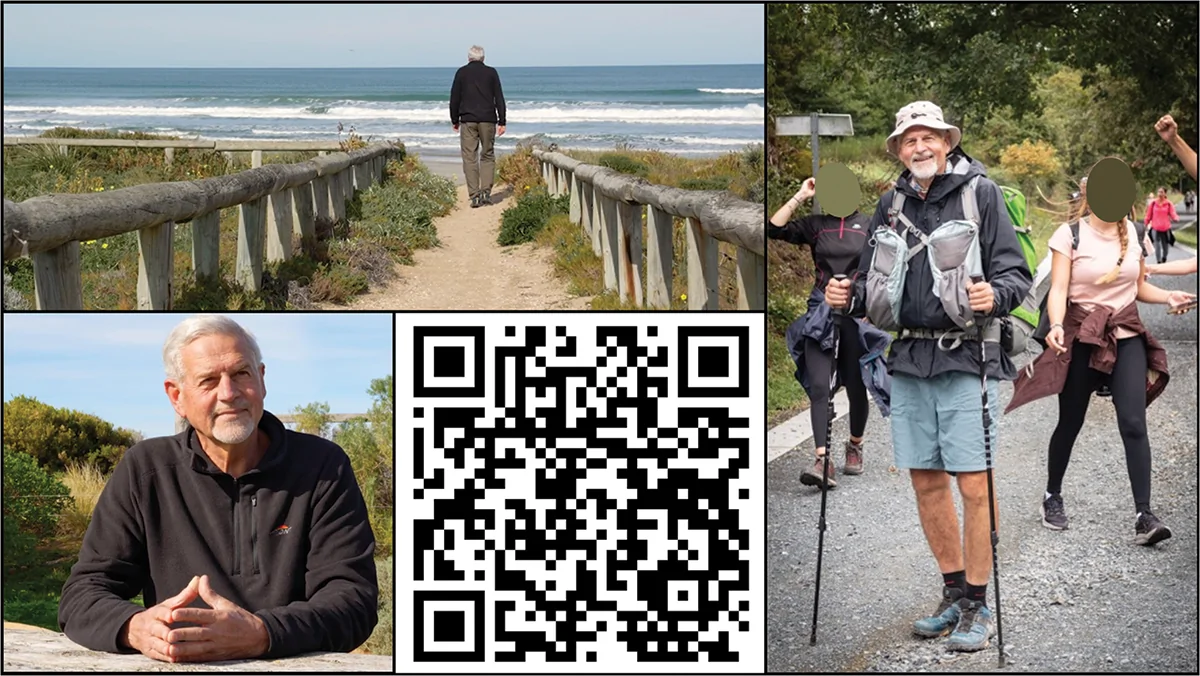
Image of PN walking the Camino de Santiago trail and screenshots from the video interview. The full video can be accessed via the QR code or the following link: https://m.youtube.com/watch?v=K8UxTXfMkIY&ra=m.

PN completed all EPIPHA-KNEE trial outcome measures at baseline, week 8, week 26, and week 52. Primary outcomes of average daily step count (via wrist-worn Actigraph GT9-X Link [Pensacola, USA]) and knee OA pain and function via the Western Ontario and McMaster Universities Osteoarthritis Index (WOMAC)^22^ are reported descriptively (raw scores, percent change from baseline) in the text. Consistent with the EPIPHA-KNEE trial, several other clinical outcomes were also evaluated and are reported in Supplementary File 3.

## Results

At trial enrollment, PN reported moderate-to-high knee pain and disability; he rated his knee pain to be an average of 6/10 overall in his right knee and recorded a WOMAC score of 45. He was also experiencing frequent episodes (2–3x per week) of knee swelling, which typically occurred after walking on the beach or prolonged standing. At baseline PN was moderately active, walking 8,133 steps per day on average, however, he was struggling to maintain this level of activity due to knee pain and swelling, and found that his knee OA was impacting his physical and mental health. “*I found that exercise or walking long distances was causing me some problems, particularly in terms of knee swelling. And the relationship between physical health and mental health as we know is very strong, so you start to feel not so great about yourself … it’s not such a good story.”*

In Session 1, the physiotherapist focused on discussing peripheral and central changes associated with persistent pain (concept 2), emphasizing that PN was safe to move (concept 1), and that physical activity is an essential OA treatment (concept 5). The physiotherapist also discussed learning strategies (concept 6). These target concepts were reinforced in Session 2, where the physiotherapist also introduced bioplasticity (concept 4) and discussed the role of systemic inflammation in knee OA (concept 1). PN reported that updating the way he conceptualized his knee OA from a degenerative condition of the knee, to understanding the benefits of weight bearing exercise for his knee joint (concepts 1, 4, and 5) held the most benefit to him. *“We often hear about [knee OA as being] bone-on-bone … which conjures up images of grinding bones. it’s all quite negative …. So instead, the concept of doing exercise, particularly load-bearing exercises, in order to have healthy cartilage really appealed to me. It really puts a positive spin on the whole idea of doing exercise.”’* This represents a shift in the conceptualization of knee OA from an unchangeable structural entity to a changeable process.

Concept 3 (pain and other protective processes are influenced by bio-, psycho-, and social contexts), was covered in Sessions 2–4. PN initially found this concept to be less relevant to his personal circumstances. However, in Session 4 of the intervention, PN identified the way he and other people in his life talked about his knee (his “dodgy” knee, affectionately using the term “cripple”), as being a Danger in Me (DIM; i.e., a personal factor identified as increasing the pain experience). The use of these negative terms reinforced the epistemic identification of knee OA as a degenerative entity. Re-framing the way that PN thought about his knee acted to reenforce his new understanding of knee OA as an emergent and changeable process. Understanding the influence of DIMs on physical activity was also useful. PN identified that walking in the city with traffic was a DIM, and that walking didn’t feel as good in this environment, compared to his usual beach walks. A Safety in Me (SIM; i.e., a personal factor identified as decreasing the pain experience) for PN was knowledge that movement was not only safe, but an essential OA treatment (concept 5). The shift of understanding exercise as a DIM to a SIM occurred during a discussion about his knee imaging findings. *“I took along my x-rays from 2017 from the orthopaedic clinic. And [physiotherapist] looked at the x-rays and said, ‘well I’m not a radiographer, but these don’t look too bad’ – these being my knees. Which I found quite heartening and that was the beginning of starting to think of coming from a perspective of my knees are giving me trouble to the realisation that these are my knees, and I’m going to look after them, and they are good …. This was the beginning of the realisation of the shift of seeing my knees as a liability to being an asset.”*

Later in the same session, PN set goals with the physiotherapist, *“I then needed to describe some sort of aspirational goal or long-term goal. And obviously without really thinking it through, or giving it much thought, I said that I would like to walk the Camino de Santiago in Spain, which is an 800 km trek … [physiotherapist] said ‘Look, there is no reason that you can’t do that’. And that was like a lightbulb moment. So, it went from a question of ‘if’ to a question of ‘when and how.’”* This moment marked a dramatic shift in PN’s thinking, given that during Session 1 he mentioned that he would like to start hiking, but that this did not seem to be a realistic option given his knee OA. Conceptual change led to sustained clinical improvement. From baseline to 52 weeks, PN had an 83% reduction in pain and a 71% improvement in his function (Figure 2). At the same time, he was walking an average of 12,122 steps per day, a 49% increase compared to baseline. Walking had become a valued activity, which had wider physical and mental health benefits. “*To me it’s [walking] has become almost like an addiction … and now if I don’t walk every morning, I really feel like something is missing.”*

**Figure 2.**
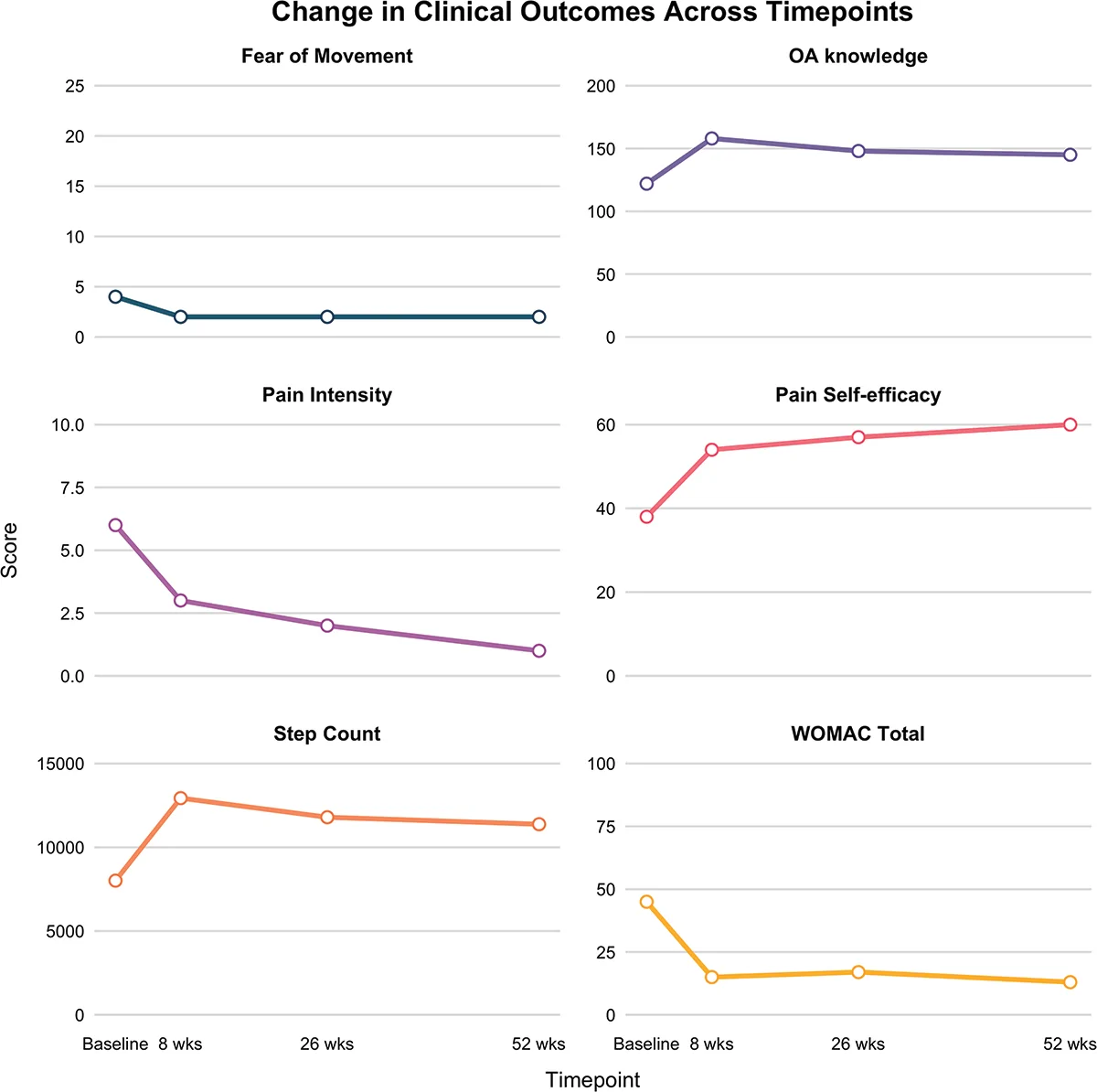
Change in clinical outcomes over the trial. WOMAC Total scores range from 0 (no symptoms or dysfunction) to 96 (maximal symptoms and dysfunction), average daily Step Count derived via wrist-worn accelerometer, Pain Intensity scores range from 0 (no pain at all) to 10 (worst pain possible), Pain Self-efficacy scores range from 0 (no confidence to perform activity despite pain) to 60 (maximal confidence to perform activity despite pain), Fear of Movement scores range from 0 (no fear of movement) to 18 (maximal fear of movement), OA knowledge scores range from 33 (conceptualisation not aligned with contemporary pain science) to 165 (conceptualisation aligned with contemporary pain science).

In 2022, two years after enrolling in the EPIPHA-KNEE trial, PN achieved his aspirational goal, walking 320 km of the Camino de Santiago trail over 14 days. Importantly, his knees did not limit his progress and he experienced no knee swelling during his walk. Although, walking the trail was not without its challenges: *“I can say that there were some pretty tough days on the Camino. I had one day that was 32 km and the last kilometre was on loose shale rock … It took me an hour to cover that one kilometre. I had to sidestep down that slope. And it was tough going. My knees were just like jelly, I would stop and there would be so many tremors. But you know what, it didn’t bother me..”*

Reflecting on his progress, PN attributes his success to multiple factors. First, his work taught him that wholistic and participatory approaches often provide the better outcomes. Such appreciation of complexity facilitated his understanding of knee OA as an emergent process. Second, PN played sport at a semiprofessional level during his youth, which gave him experience in overcoming setbacks and adversity related to pain/injuries. He enjoys challenging himself, which is reflected in the fact that once he decided to walk the Camino de Santiago, he never thought of not completing the journey as planned. Finally, he is a firm believer in setting aspirational goals that challenge the status quo, and believes that this strategy would be useful for others with knee OA, *“The important thing is to set a goal that is going to be fun, you’re going to do it on a holiday or with friends or family that you enjoy being with, and that you’re going to achieve that goal in that context.”*

As of 2024, over four years since enrolling in the study, PN continued to successfully self-manage his knee OA: *“I have to say my knees are great! I do have to say that there are days when I have pain, but I just simply manage that. I know that pain is a normal thing … the program taught me that we can change our expectations in terms of managing pain and I certainly do that.”* He is optimistic about the future and his continued ability to self-manage his knee OA: *“Right now my overall goal is to continue doing what I’m doing health wise, to look after myself, think positively about my knees, look after them, and just enjoy life.”* Reflecting on his progress, PN’s message to other people with knee OA is *“It’s to be optimistic, to have confidence in your body, and to have hope that you will be able to manage your osteoarthritis.”* If PN could change anything about his journey, it would be exploring conservative management sooner: *“Knowing what I know now, I would have embarked upon this journey a lot earlier. I would have looked into things back in 2009, when I had my unfortunate outing with tennis, which really stopped me playing tennis and really got me into a really negative way of thinking about my knees.”*

PN wants health providers to offer people with knee OA a range of management options, including conservative management, so that people with OA are informed and involved in the decision-making for their OA management: *“What I was missing initially was a broader understanding of what’s involved with joint pain and osteoarthritis, in other words going beyond the surgical solution … there are an awful lot of people being referred directly to orthopaedic surgeons and the likely result of that is surgery, that’s the way it works. So, I think having health care professionals that have a broader approach to managing osteoarthritis is key. Because once we are given options, we can make more informed decision about what we choose to do.”* In line with this message PN and the research team co-produced a video where PN tells his story of recovery (Figure 1). This video is intended to be used as a free educational resource for health care professionals and people with knee OA to demonstrate the potentially transformative impacts of conservative management of knee OA.

## Discussion

This case report demonstrates real-world, life-altering outcomes from a PSE and graded exercise program for knee OA. PN had a long history of OA-related knee pain and was advised that his only treatment option was knee arthroplasty. PN’s decision to instead participate in the EPIPHA-KNEE trial resulted in marked improvements in knee pain, function, and physical activity levels. Sustained improvements allowed PN to achieve his ‘aspirational goal’ to walk the Camino de Santiago Trail. Currently, four years later, PN continues to self-manage his knee OA using strategies he developed during the trial, including daily walking. This indicates that sustained self-management of knee OA symptoms is possible, a result that has not been clearly demonstrated in the extant literature.^23^ Alongside the case study, we provide clinical resources, including a brief video of PN telling his story. We anticipate that these resources could hold benefit for patient education as an authentic demonstration of the benefits of exercise, PSE, and self-management in improving knee OA outcomes, as well as provide an exemplar of what can be achieved regardless of OA severity shown on a scan.

Our video showing PN’s journey fills an unmet need. People with knee OA consider case studies and testimonials as powerful sources of evidence and are perceived as more reliable than numbers or statistics provided by randomized controlled trials or meta-analyses.^20^ This is in contrast to typical evidence hierarchies favored by researchers and clinical-practice guidelines.^24,25^ Indeed, discrepancies between the types of evidence prioritized by researchers, health care professionals, and people with knee OA may in part underlie the translational failures of knee OA research to clinical practice. Health decisions are complex and often require patients and health care professionals to evaluate the benefits and risks of more than one treatment option. Typically, this information is presented numerically and in terms of group-level efficacy, which may not be optimal, as statistics can be confusing for both people with OA and health care professionals.^26^ Instead, narratives have been shown to communicate information more effectively than more didactic tools (e.g., patient decision aids).^27^ When provided together, consumers favor anecdotal evidence over peer-reviewed research, even when the anecdotal evidence is inaccurate and not supported by research.^28^ There is merit in providing *accurate narrative evidence* derived from high-quality case studies. The wider literature would support the value of PN’s narrative video in assisting health outcomes. There is growing evidence for the power of narrative storytelling across several major public health issues, including sexual health, substance abuse, and vaccinations.^28,29^ Currently, typical OA discourse is impairment focused,^30^ with both negative explicit (i.e., joint wear-and-tear) and implicit (i.e., direct referral to a surgeon) messaging. Here, PN identified with positively framed messages, and found SIMs more personally relevant than DIMs. Indeed, the physiotherapist telling PN that he could walk the Camino de Santiago Trail represented a turning point in his OA recovery and acted as a highly motivating SIM. Positively framed messaging may be particularly important to include in future OA educational materials.

Providing personal narratives may also be a particularly powerful tool to optimize the delivery of PSE, given evidence that some patients find PSE concepts to be irrelevant to their personal situation or even find some concepts problematic.^31,32^ Traditional, didactic PSE interventions are increasingly recognized as suboptimal^33^ and can be invalidating or stigmatizing to some people living with persistent pain. Indeed, people with chronic pain report negative attitudes toward PSE statements (e.g., pain is not an accurate indicator of tissue damage) within a social media context.^32^ PSE interventions can be challenging for some people living with persistent pain, as concepts may be in opposition to deeply held beliefs about one’s own body (e.g., that knee pain during exercise is due to wear-and-tear of knee joint structures). Inaccurate beliefs about pain are often reenforced within a person’s social environment, including their peers or even their health care professionals.^34^ Personal narratives of a peer with shared experience may be a more acceptable, credible, and validating way for inaccurate body-related beliefs to be challenged, or work through alternate mechanisms (e.g., empathetic connections, cultural embeddedness).^35^ There is merit in exploring the efficacy of storytelling videos, such as the video provided here, in delivering PSE and inducing behavioral change in people with knee osteoarthritis. One can also consider contemporary PSE as an intervention characterized by reciprocal and progressive storytelling (i.e., the patient sharing their story with their clinician, the clinician sharing stories to help make sense of pain). Here, by reframing his personal OA story, PN was able to adjust his goals overtime, ultimately setting and achieving his aspirational goal of walking the Camino Trail.

Given case study evidence, there are important limitations to consider. Without a comparator, it is uncertain the extent to which observed effects are due to the PSE/graded exercise intervention or nonspecific treatment effects (i.e., natural history or therapist connection). However, changes in physical activity and knee OA/physical activity beliefs were temporally linked (i.e., greatest changes occurred during the intensive 8 weeks of the program, which were maintained at all subsequent timepoints), which supports that the intervention itself (specific and nonspecific effects combined) was effective. Further, attenuated long-term benefits of physical activity for OA has been shown to be related to a reduction in activity levels,^36^ providing support for PN’s sustained clinical improvement given sustained long-term activity levels. Knee OA is a progressive condition,^37^ thus the sustained clinical benefit (over years) seen here are in *opposition* to the expected trajectory of knee OA.

Generalizability of these findings to other people with painful knee OA is unknown. PN is educated, of high socioeconomic status, and has high health literacy. Having the ability to walk the Camino Trail itself is a privilege (i.e., requires money, time, and travel), regardless of one’s knee OA status. It is therefore unknown if people of diverse educational or socioeconomic backgrounds would derive similar benefits from the intervention described here, nor if they would identify with PN’s story. Upward comparisons with the narrator of a story can be beneficial (e.g., that person is managing their OA well, maybe I could do the same).^38^ However, if the upward comparison is too high, then the story may be disregarded altogether or even generate negative feelings (e.g., that is impossible, there is no way I could achieve that with my knees). Given PN’s remarkable achievements, there is risk of the latter, thus, clinicians should be mindful of the way that they frame PN’s story to people with knee OA to mitigate potential unfavorable comparisons. It is also important to consider that across the literature, health education interventions show reduced effectiveness in those from disadvantaged socioeconomic backgrounds,^39^ therefore this case study may not generalize to these groups. Caution is also warranted in setting aspirational goals; clinicians should work with patients to choose goals that are ultimately *achievable for each individual* (i.e., are financially or practically possible). Unfulfilled expectations have been implicated in poor knee OA outcomes, particularly post arthroplasty.^40^ Thus, clinicians must balance optimism with reality when setting aspirational goals. Finally, while the primary and secondary outcomes reported here are consistent with the EPIPHA-KNEE trial, the specific analyses were not pre-registered and should be interpreted with caution.

## Conclusion

This case study aims to highlight the real-world, life-changing benefits of non-surgical management of knee OA. PN was able to achieve his aspirational dream of walking the Camino de Santiago Trail five years after being told his only remaining management option was joint arthroplasty. He was empowered to continue to self-manage his knee OA independently, supporting that engaging in an individualized graded exercise program and updating one’s understanding of their OA can elicit and sustain long-term behavioral change. We provide clinical resources aimed at both clinicians and those living with knee OA. In capturing PN’s story in his own words using a digital story, we hope that this case provides knee OA consumers and health professionals with a useful resource, namely, an inspirational example of what can be achieved with best-practice knee OA management.

## Supplementary Material

Supplementary files.docx
