# Supplementary material for: “I Was Told I Needed Both Knees Surgically Replaced, but I Walked the Camino Trail Instead.” A Case Report of A 66-year-old Male with Knee Osteoarthritis
Source: Can J Pain. 2026 Sep 29;10(1):2717606. doi: 10.1080/24740527.2026.2717606 (PMC13625712; doi:10.1080/24740527.2026.2717606)
Supplement: Supplementary file 1 [file UCJP_A_2717606_SM6659.docx]

**Supplementary File 1**

**Supplementary File 2 – deviations from published protocol and Interview questions**

| **Deviation/error** | **Rationale** |
| --- | --- |
| First follow-up at 8 weeks, instead of 52 weeks | This change was made for the full trial after piloting, as this allowed for the completion of the entire block of 8 weekly sessions prior to follow-up. |
| Several of the lab-based outcomes (evaluated at baseline and 8 weeks) are not reported here | Specific reasons for each outcome measure:  - Implicit association task: still under development during piloting and was not completed by PN  - Distance/hill steepness estimation task: still under development during piloting and was not completed by PN  - 2-point discrimination: evaluated by two different assessors across timepoints. Unknown reliability of these assessors limits interpretation, thus not reported  - Left-right judgement task: was not completed at baseline. |
| EQ VAS | At baseline, the anchors on this scale were reversed. However, this looked to be answered correctly by PN, thus we included this measure in the current study |
| Addition of sleep analysis | This additional analysis was performed as PN reported disrupted sleep at baseline, which improved during the trial. |

**Semi-structured interview guide**

**Defining the event**

Tell me your story – when did your problems with your knee begin?

How did your knee pain impact your life?

Before the trial what had you tried to help your knees?

Tell me about the appointment with the surgeon – what did you take away from that experience?

Tell me about the lead up to the trial – what lead you to sign up for it? Did you have any expectations going into it?

**Key moments**

- Appointment with the surgeon
  - Surgery presented as the only option
  - Where to next? No information about other management strategies
- Friends bad experience with knee replacement

Extra questions (if needed)

1. When did you first begin having problems with your knees?
2. Were your knee problems related to a specific incident (sporting injury) or did it seemingly come on for no reason?
3. How did your knee pain impact your life?
   a. Exercise, work, family and plans for the future
4. When were your knees at their worst?
   a. At that time, what did you think was causing this?
5. Who did you see (health professional) for your knees?
   a. What triggered you to seek out help? Was there one moment that motivated you to book the appointment?
6. What testing did they do and what were the results (diagnosis)?
   a. What were the x-ray results and how did it feel to get those results
7. What advice did they (health professional) give?
   a. How did it feel to hear the surgeon say that it was all downhill from here? How did you respond to that information?
8. What treatment(s) did you undergo and what were the results?
   a. What medications did you try and what were the results?
9. Why did you decide to participate in the clinical trial?
   a. Was there one moment that made you decide that you wanted to do things differently?
10. Going into an exercise trial – what did you expect would happen?
    a. Did you actually believe that your knees would get better with exercise?

**Defining the turning point**

Tell me about your experience with Kim and the physio program.

Tell me about the first time you discussed walking the Camino with Kim – how did that conversation go?

Once you set your aspirational/Camino goal – what then? How did you go about achieving it?

Tell me about the Camino – what was that experience like?

**Key moments**

- Kim’s reaction to the x-rays – the lightbulb moments
- The intervention – working with a physio who knows what they are doing – mutual accountability
- Education – key new information
- How the lead up to the trial impacted things

Extra questions (if needed)

1. Describe your experience of the program/physio, including what you learnt, what you achieved, and how your knees responded (symptoms).
2. Tell us about your first physio session with Kim – what was your first impression of the program?
3. What was the most surprising thing that you learnt about knee OA?
   a. What was the most impactful thing you learnt?
4. What did you feel was the most difficult part? Did you have any setbacks?
   a. If so, can you tell us about it and how you overcame it?
5. What do you attribute your improvement to?
6. When were your knees at their best? What do you believe contributed to your knees being at their best during this time?
7. How did you find training for the Camino?
   a. What did this look like and how did you keep on track?
8. What was your day to day like on the Camino?
   a. Were there any particularly tough days?
   b. Were there any standout days – where everything just fell into place?

**Defining the happy ending – the ongoing good news story!**

Achieving something is one thing – but then what next? What drives/motivates you to keep going long term?

What are you doing these days and how is your knee?

What would do you most want other people with OA to know?

Knowing what you do now, would you change anything about how you initially managed your knee pain?

Extra questions (if needed)

1. How did it feel to finish the Camino?
2. How are your knees now?
   a. How do you deal with flare ups?
3. What’s next? Do you have any new goals?
4. Knowing what you do now, would you change anything about how you initially managed your knee pain?
5. What would do you most want other people with OA to know
   a. There is hope
6. Achieving something is one thing – but then what next? What drives/motivates to keep going long term
   a. Internal motivator – if you feel good, why stop doing it?

**Supplementary File 3 – clinical and experimental outcomes**

Table 1: Clinical and experimental outcomes

|  |  | Baseline | Week 8  (% change from baseline) | Week 26  (% change from baseline) | Week 52  (% change from baseline) |
| --- | --- | --- | --- | --- | --- |
| **Primary outcomes** |  |  |  |  |  |
| WOMAC | Total score | 45 | 15 (↓67%) | 17 (↓62%) | 13 (↓71%) |
|  | Pain subscale | 9 | 2 (↓78%) | 2 (↓78%) | 1 (↓89%) |
|  | Stiffness subscale | 5 | 3 (↓40%) | 4 (↓20%) | 2 (↓60%) |
|  | Physical function subscale | 31 | 10 (↓70%) | 11 (↓65%) | 10 (↓70%) |
| Average Step count |  | 8,133 | 11,604 (↑43%) | 13,363 (↑64%) | 12,122 (↑49%) |
| **Secondary outcomes** |  |  |  |  |  |
| Average knee pain intensity over the past week (0-10 NRS) | Overall | 6 | 3 (↓50%) | 2 (↓67%) | 1 (↓83%) |
|  | While walking | 5 | 3 (↓40%) | 3 (↓40%) | 1 (↓80%) |
| Pain self-efficacy (PSEQ) |  | 38 | 54 (↑42%) | 57 (↑50%) | 60 (↑58%) |
| Health-related quality of life (EQ-5D-5L) | Health state code | 32231 | 11221 | 11121 | 11211 |
|  | EQ VAS | 80 | 80 (0%) | 90 (↑13%) | 85 (↑6%) |
| Depression (D-PROMIS) |  | 0 | 0 (0%) | 0 (0%) | 0 (0%) |
| Anxiety (A-PROMIS) |  | 0 | 0 (0%) | 0 (0%) | 0 (0%) |
| Stress (PSS) |  | 1 | 1 (0%) | 0 (↓100%) | 0 ↓00%) |
| Fear of movement (BFoM) |  | 4 | 2 (↓50%) | 2 (↓50%) | 2 (↓50%) |
| Pain catastrophising (B-PCS) |  | 3 | 0 (↓100%) | 1 (↓67%) | 5 (↑67%) |
| Knee perception (FREKAQ) |  | 9 | 3 (↓67%) | 5 (↓44%) | 1 (↓89%) |
| OA Concepts | Total score | **122** | 158 (↑30%) | 148 (↑21%) | 145 (↑19%) |
|  | OA concepts | **76** | 101 (↑33%) | 91 (↑20%) | 91 (↑20%) |
|  | Physical activity concepts | **46** | 47 (2%) | 57 (↑24%) | 54 (↑17%) |
| Self-regulated learning (SRL) |  | 43 | 49 (↑14%) | 49 (↑14%) | 49 (↑14%) |
| Global rating of change (GRC) |  | – | 6 (↑) | 10 (↑) | 9 (↑) |
| Average daily minutes of (unbouted) activity | Sedentary | 716 | 765 (↑6%) | 692 (↓3%) | 745 (↑5%) |
|  | Light | 126 | 142 (↑13%) | 157 (↑25%) | 108 (↓14%) |
|  | Moderate | 63 | 105 (↑67%) | 136 (↑116) | 120 (↑90%) |
|  | Vigorous | 2 | 7 (↑385%) | 2 (0%) | 5 (↑267%) |
| Sleep analysis | Sleep duration (hours) | 7.40 | 8.00 (↑8%) | 7.14 (↓4%) | 7.43 (↑0%) |
|  | Number of awakenings | 16 | 16.7 (↑4%) | 16.7 (↑4%) | 14.8 (↓8%) |
|  | Sleep regularity index | 46 | 43 (↓7%) | 23 (↓49%) | 34 (↓28%) |
| Medications |  | Paracetamol  Topical anti-inflammatory | None | None | None |
| Pain sensitivity to activity (6 minute walk test) | Change in pain (pre-post walk) | +10 | +10 (0%) |  |  |
|  | Distance walked (metres) | 320 | 542 (↑69%) | – | – |
| **Process outcomes** |  |  |  |  |  |
| Walking Adherence Questionnaire (WAQ) | Adherence | – | 30 | 30 | 30 |
|  | Number of days walked for exercise in the past week | – | 7 | 7 | 7 |
| Strengthening exercise Adherence Questionnaire (SAQ) | Adherence | – | 20 | 10 | 16 |
|  | Number of days exercises were performed in the past week | – | 2 | 2 | 1 |
| Intention to Exercise (ITE) |  | – | 18 | 18 | 18 |

Improvement from baseline; Deterioration from baseline; ↓ decrease in score; ↑increase in score;– not assessed.

WOMAC Western Ontario McMaster Universities OA Index (pain subscale: 5 items, 0-4 Likert scale, score range 0-20, lower score represents lower pain levels; physical function subscale: 17 items, 0-4 Likert scale, score range 0-68, lower score represents less difficulty; stiffness subscale 2 items, 0-4 Likert scale, score range 0-8, total WOMAC score is the sum of the 3 subscales, maximum score 96);

NRS Numeric Rating Scale for pain (score range 0-10, lower score represents less pain);

PSEQ Pain Self-Efficacy Questionnaire (10 items, 0-6 Likert scale, score range 0-60, lower score represents lower confidence);

EQ-5D-5L EuroQOL 5 level instrument (health state subscale: 5 items, 1-5 Likert scale, 5 individual ratings coded into a 5-digit number; EQ Visual Analogue Scale (VAS), score range 0-100, lower score represents poorer health);

D-PROMIS Depression-Patient Reported Outcomes Measurement Information System (4 items, 0-4 Likert scale, score range 0-15, lower score represents less depression);

A-PROMIS Anxiety-Patient Reported Outcomes Measurement Information System (4 items, 0-4 Likert scale, score range 0-15, lower score represents less anxiety);

PSS Perceived Stress Scale (4 items, 0-4 Likert scale, score range 0-16, lower score represents less stress)

BFoM Brief Fear of Movement Scale (6 items, 0-3 Likert scale, score range 0-18, lower score represents less fear);

B-PCS Brief Pain Catastrophizing Scale (4 items, 0-4 Likert scale, score range 0-16, lower score represents less catastrophizing);

FREKAQ Fremantle Knee Awareness Questionnaire (9 items, 0-4 Likert scale, score range 0-36, lower score represents less perceptual dysfunction);

OACQ Osteoarthritis Conceptualisation Questionnaire (OA concepts subscale: 20 items, 0-4 Likert scale, score range 0-80; physical activity concepts subscale: 13 items, 0-4 Likert scale, score range 0-52; total OACS score is the sum of the 2 subscales, score range 0-132, lower score represents lower conceptualisation);

SRL Self-Regulated Learning (7 items, 1-7 Likert scale, score range 7-49, lower score represents less SRL);

GRC Global Rating of Change (4 items, scale from -3 to +3, score range -12 to +12, negative score represents worsened, positive score represents improved);

Medications Taken at least once per week over the last month;

Pain sensitivity to activity Knee pain intensity ratings (NRS, score range 0-100, lower score represents less pain) at each minute during a standardised 6-minute walk test. The distance walked is also reported.

WAQ Walking Adherence Questionnaire (3 items, 0-10 NRS, score range 0-30, lower score represents less adherence);

SAQ Strengthening exercise Adherence Questionnaire (2 items, 0-10 NRS, score range 0-20, lower score represents less adherence);

ITE Intention to Exercise (3 items, 0-6 Likert scale, score range 0-18, lower score represents less intention to exercise);

COVID-19 impact on p
